# Characterization of a Novel Enterovirus Serotype and an Enterovirus EV-B93 Isolated from Acute Flaccid Paralysis Patients

**DOI:** 10.1371/journal.pone.0080040

**Published:** 2013-11-07

**Authors:** Shahzad Shaukat, Mehar Angez, Muhammad Masroor Alam, Salmaan Sharif, Adnan Khurshid, Tariq Mahmood, Syed Sohail Zahoor Zaidi

**Affiliations:** 1 Department of Virology, National Institute of Health, Chak Shahzad, Islamabad, Pakistan; 2 Department of Biotechnology, Faculty of Biological Sciences, Quaid-i-Azam University, Islamabad, Pakistan; 3 Department of Plant Sciences, Faculty of Biological Sciences, Quaid-i-Azam University, Islamabad, Pakistan; Columbia University, United States of America

## Abstract

Non-polio enteroviruses (NPEVs) are among the most common viruses infecting humans worldwide. Most of these infections are asymptomatic but few can lead to systemic and neurological disorders like Acute Flaccid Paralysis (AFP). Acute Flaccid Paralysis is a clinical syndrome and NPEVs have been isolated frequently from the patients suffering from AFP but little is known about their causal relationship. The objective of this study was to identify and characterize the NPEV serotypes recovered from 184 stool samples collected from AFP patients in Federally Administered Tribal Areas (FATA) in north-west of Pakistan. Overall, 44 (95.6 %) isolates were successfully typed through microneutralization assay as a member of enterovirus B species including echovirus (E)-2, E-3, E-4, E-6, E-7, E-11, E-13, E-14, E-21 and E-29 while two isolates (PAK NIH SP6545B and PAK NIH SP1202B) remained untypeable. The VP1 and capsid regions analysis characterized these viruses as EV-B93 and EV-B106. Phylogenetic analysis confirmed that PAK NIH isolates had high genetic diversity and represent distinct genotypes circulating in the country. Our findings highlight the role of NPEVs in AFP cases to be thoroughly investigated especially in high disease risk areas, with limited surveillance activities and health resources.

## Introduction

Human enteroviruses (HEVs) are small, non-enveloped, positive sense, single-stranded RNA viruses belonging to the family *Picornaviridae* [1]. Recently, HEVs are classified into seven species; Enterovirus (EV)-A, EV-B, EV-C, EV-D, Rhinovirus (RV)-A, RV-B and RV-C [2]. HEVs are responsible for a wide range of diseases in human worldwide including cutaneous, visceral and neurological disorders. The neurological disorders range from aseptic meningitis to brainstem encephalitis and Acute Flaccid Paralysis (AFP) [1,3,4]. Although poliovirus is considered as a major cause of AFP, many non-polio enteroviruses (NPEVs) cause persistent flaccid paralysis [5-12]. Therefore, the evaluation of transmission of NPEVs in the community is one of the major factors for determination of their role as non-polio AFP etiologies. 

**Figure 1:**
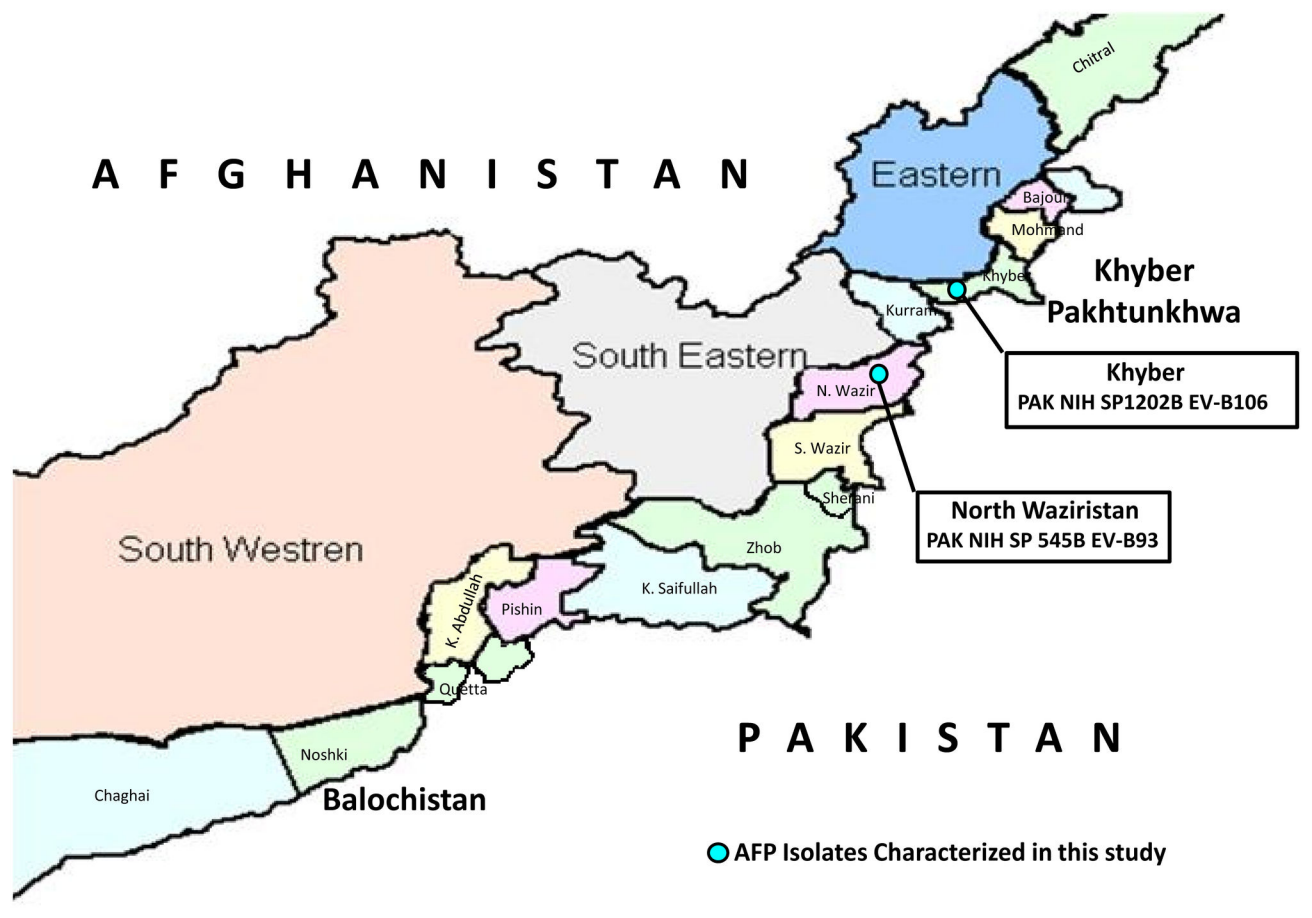
Map of the Federally Administered Tribal Areas (FATA) in north-west of Pakistan bordered by: Afghanistan to the north and west with the border marked by Khyber Pakhtunkhwa to the east and Balochistan to the south. The symbol ‘●’ shows the geographical positions of the acute flaccid paralysis isolates characterized in this study through molecular sequencing.

In Pakistan, no enterovirus surveillance system is in place. Therefore, no reliable data about the diseases burden of NPEVs in the community and hospitalization is available. Moreover, Pakistan is still a polio endemic country and because of its greater public health importance, other non-polio enteroviruses are rarely studied. The present study specially focuses on the identification and characterization of NPEVs isolated from AFP cases residing in the Federally Administered Tribal Areas (FATA). Geographically it is a tribal region in the north-western Pakistan having seven tribal agencies in order from north to south: Bajaur, Mohmand, Khyber, Orakzai, Kurram, North Waziristan and South Waziristan (Figure 1). More importantly, over the past decade, war against terrorism and militancy in this region are main factors for the migration of families across the country. This unnoticed movement of internally displaced persons in other parts of the country explores the challenges for public health that is needed for investigation. 

The current study reports the identification of twelve enterovirus serotypes including recently identified EV-B106 and a more distinct genotype of EV-B93 from stool of children suffering from AFP. Our findings highlight that the transmission of divergent enterovirus strains in specific population group can lead to increase in paralytic cases and may complicate the evaluation of poliomyelitis control strategies. 

## Materials and Methods

### Ethics Statement

This study was approved by Institutional Internal Review Committee. Written informed consent was obtained from all the individuals participated. A check box was included in data form to document the consent taking procedure.

A total of 184 stool samples of AFP children under < 15 years of age were collected from FATA during 2010. Samples were processed at virology Department, National Institute of Health, Islamabad, Pakistan. The NPEVs isolated on RD (Human rhabdomyosarcoma) and L20B (Mouse fibroblast cells expressing with poliovirus receptor) cells were characterized by microneutralization assay according to WHO laboratory protocol using RIVM antiserum pool [13]. The untypeable isolates were confirmed as NPEV through real-time PCR [14] after extracting the RNA using QIAamp viral RNA mini kit (Qiagen). All untypeable NPEV isolates were subjected to reverse transcriptase PCR for VP1 gene sequencing using forward primer 490 (5´-TGI GTI YTI TGY RTI CCI TGG AT-3´) and reverse primer 493 (5´-TCN ACI ANI CCI GGI CCY TC-3´) [15]. The amplicons were purified and sequencing reactions were performed using Big Dye Terminator Ready Reaction Mix (Version 3.1, Perkin Elmer Applied Biosystems) and were sequenced in an automated ABI 3100 genetic analyzer. Sequences were edited using Sequencher software version 4.9 (Gene Codes Corporation, USA) and blasted online using blastn and tblastx algorithms (http://www.ncbi.nlm.nih.gov/BLAST). Sequences were aligned by ClustalW [16] with the representative enterovirus sequences retrieved from GenBank. The distance matrix was calculated using Kimura 2 parameter [17] and Phylogenetic tree was constructed by Neighbor joining method using MEGA version 5.0 software. Treeview version 1.5.3 [18] was used to display the evolutionary tree and bootstrap analysis was performed with 1000 pseudo replicates. Any isolate showing <75% nucleotide (<82% amino acid) identity in VP1 gene to the sequence of known enterovirus type was flagged as a new enterovirus type and the isolate showing <85% nucleotide homology with prototype strain was confirmed as its new genotype [19-21].

The nucleotides sequences of the isolates characterized in this study are submitted in GenBank under accession no KF385943 and KF385945.

## Results

**Figure 2:**
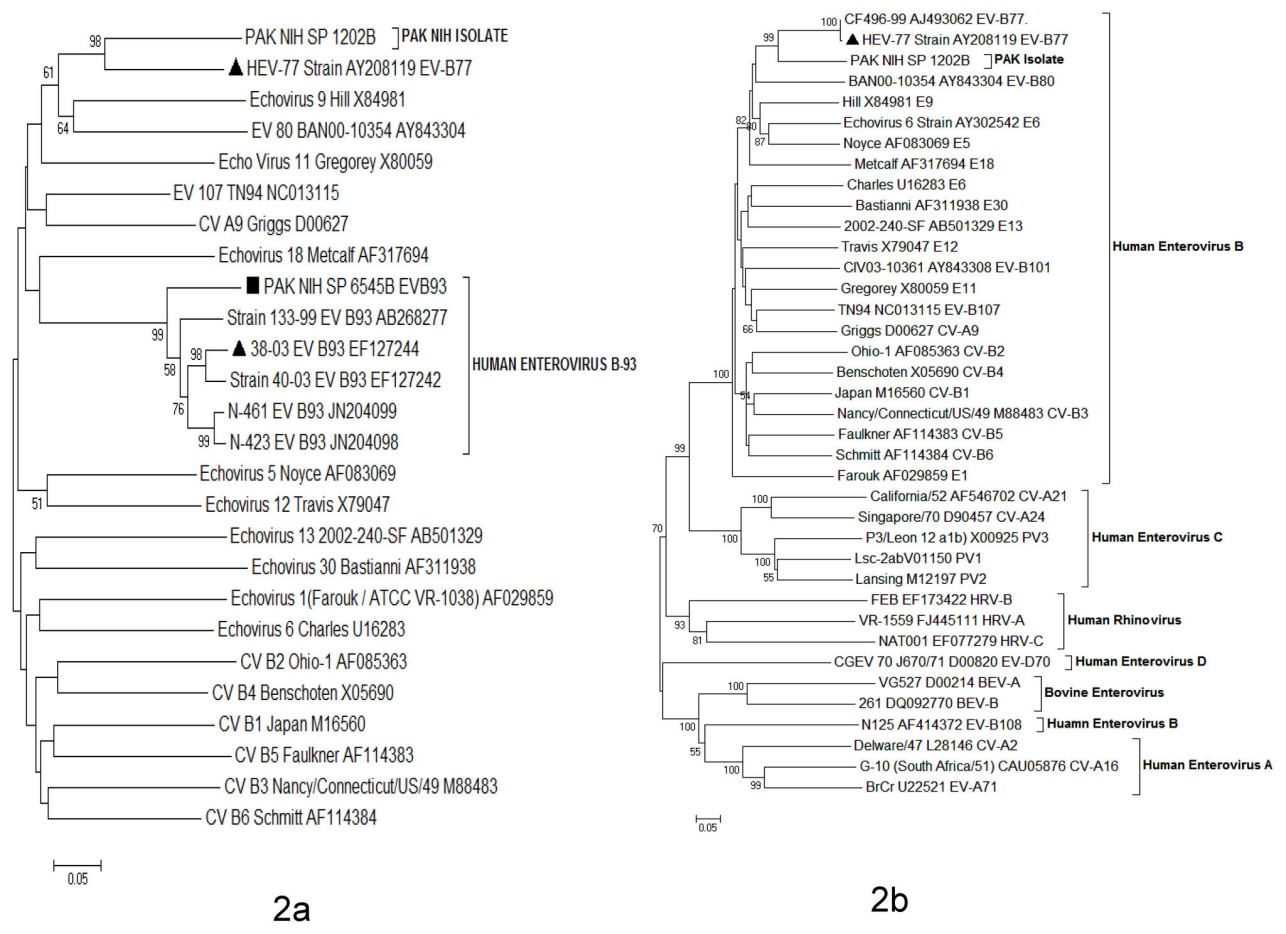
Unrooted Phylogenetic tree of Human Enteroviruses was constructed by Neighbor-joining (NJ) method with Kimura 2-parameter (K2-P) model using MEGA version 5.0 software. This Phylogenetic tree based on A) VP1 and B) whole capsid (VP4-VP1) nucleotide sequences of PAK NIH isolates and other representative sequences of enterovirus serotypes retrieved from GenBank. AFP isolate is represented by ‘■’ taxon marker. The Prototype strain is labeled with taxon marker ‘▲’. Tree was evaluated with 1000 bootstrap pseudoreplicates. Bootstrap values greater than 50 are indicated at the respective nodes and the scale bar represents the evolutionary distance.

Overall, 46 (25%) stool samples were found positive for NPEV, 63 (34.2%) for poliovirus and 75 (40.8%) remained negative on cell culture. By neutralization assay, 44 isolates (95.6%) were typed into ten serotypes including echovirus (E)-2 (2.2%), E-3 (4.3%), E-4 (2.2%), E-6 (8.7%), E-7 (10.9%), E-11 (13.0%), E-13 (17.4%), E-14 (13.0%), E-21 (6.5%) and E-29 (17.4%) while two (4.4%) isolates (PAK NIH SP1202B and PAK NIH SP6545B) remained untypeable. Clinical findings, symptoms at onset of disease and other epidemiological details of non-polio enteroviruses were collected and summarized (Table 1). The untypeable isolates were subjected to real-time PCR for confirmation of NPEV and characterized by sequencing of VP1 and capsid regions (VP4, VP2, VP3 and VP1). PAK NIH SP1202B isolate was found closely related to prototype strain (Lafeuille-W543-122/99; AY208119) of EV-B77 having 73.4% nucleotide (83.5% amino acid) identity in VP1 region while it had 65.5% mean nucleotide (68% mean amino acid) identity with representative members of EV-B species. According to the enterovirus type demarcation criteria (<75% nucleotide or <88% amino acid homology with known members across the VP1 sequence), it is proposed that PAK NIH SP1202B isolate belongs to a new serotype. Pairwise comparison of the whole capsid region further confirmed that this isolate had 77.2% nucleotide (86% amino acid) identity with its closely related strain of EV-B77 (Lafeuille-W543-122/99; AY208119) and 67.8% (72.8% amino acid) mean nucleotide identity with representative members of EV-B species (Figure 2). Similarly, on comparison with representative members of EV-A, EV-C, EV-D, BEV and HRV species mean nucleotide and amino acid identities were 51.5% (44.5% amino acid), 56.5% (55.8% amino acid), 53.1% (41% amino acid), 51.5% (43% amino acid) and 51.8% (40% amino acid) respectively. On submission of VP1 nucleotide sequence of PAK NIH SP1202B isolate to International Committee on Taxonomy of Viruses (ICTV), they assigned it as EV-B106 serotype, however, since no reference sequence of EV-B106 is available in GenBank, we were not able to confirm this classification. 

**Table 1:** Clinical and Virological Findings in Acute Flaccid Paralysis Patients (n=46) having Enterovirus Infections.

	**E-02**	**E-03**	**E-04**	**E-06**	**E-7**	**E-11**	**E-13**	**E-14**	**E-21**	**E-29**	**EV-B93**	**EV-B106**
	**n=1**	**n=2**	**n=1**	**n=4**	**n=5**	**n=6**	**n=8**	**n=6**	**n=3**	**n=8**	**n=1**	**n=1**
**Mean Age (months)**	20	16	12	11	8	6	14	20	9	17	19	18
**Sex (male/female)**	0/1	1/1	0/1	3/1	3/2	4/2	5/3	3/3	1/2	3/5	0/1	0/1
**Fever at onset of paralysis No. (%)**	1 (100)	2 (100)	1 (100)	3 (75)	4 (80)	5 (83)	6 (75)	4 (66)	1(100)	6 (75)	1 (100)	1(100)
**Asymmetrical Paralysis No. (%)**	1 (100)	2 (100)	1 (100)	2 (50)	3 (60)	3 (50)	6 (75)	4 (66.6)	1 (33)	5 (62.5)	1 (100)	1 (100)

E: Echovirus; EV: Enterovirus

Furthermore, phylogenetic analyses of the capsid region revealed that PAK NIH SP1202B clustered with EV-B77 strain having 98 as bootstrap value (Figure 2). The epidemiological data showed that PAK NIH SP1202B was isolated from the stool of 18 months old AFP child residing in Khyber Agency (Figure 1). This child had high-grade fever, asymmetric paralysis in her right lower limb, flaccid tone, loss of reflexes and showed progression during neuromuscular examination. Electromyography (EMG) has also confirmed these findings. The expert review committee for classifications of AFP cases categorized it as an enterovirus neuropathy case. Furthermore, persistent residual weakness of right lower limb was observed at 60 days follow- up of this child. 

The partial VP1 nucleotide sequence (597bp) of PAK NIH SP6545B isolate had 84.1% nucleotide (93.4% amino acid) identity with EV-B93 prototype strain (EF127244) (Figure 2). PAK NIH SP6545B strain was isolated from a 19 months old child suffering from AFP who resided in a border area of North Waziristan (Figure 1). She had high grade fever, loss of muscular tone in the left lower limb and was preliminary diagnosed as poliomyelitis. sixty days post follow-up was not available for this patient. 

## Discussion

Isolation of NPEVs from AFP cases is common worldwide [22-28] but clinically it is difficult to determine their association with paralytic disorders. However, few NPEVs including enterovirus 70 and 71; coxsackievirus type A4, 6, 7, 9, 11, 14 and 21; coxsackievirus type B1, 2, 3, 4, 5 and 6 and echovirus type 1, 2, 3, 4, 6, 7, 9, 11, 16, 18, 19 and 30 have been reported to cause this crippling disorder [5,29-31]. In Pakistan, no routine surveillance of NPEVs is in place but their isolation is documented only to monitor the sensitivity of the national polio eradication surveillance program. 

Non-polio enterovirus strains having <75% nucleotide or <88% amino acid identity in VP1 are classified into different serotypes as proposed by Oberste et al. (1999) [32]. In our study, molecular phylogenetic analysis based on the VP1 gene showed a significant divergence of untypeable NPEV strains when compared to their prototypes. PAK NIH SP1202B isolate has >25% sequence diversity in both VP1 and capsid regions (VP4-VP1) when compared to the prototype strain of EV-B77 (Lafeuille-W543-122/99; AY208119). Based on the standard demarcation criteria for classifying enterovirus serotypes [32], our data indicates that PAK NIH SP1202B is a new serotype of enterovirus B species. Due to lack of available reference sequence in GenBank, the VP1 sequence of PAK NIH SP1202B was submitted to ICTV. Their analysis re-assured PAK NIH SP1202B as an enterovirus serotype; EV-B106. 

Similarly, PAK NIH SP6545B isolate clustered separately with known EV-B93 strains and showed >15% divergence in VP1 gene to EV-B93 prototype strain (Strain 38-03; EF127244), possibly indicating a new genotype of EV-B93 circulating in FATA. 

These viruses were isolated from the patients having asymmetrical paralysis with rapid progression and high-grade fever at the time of onset. These symptoms resemble to the cardinal signs of poliomyelitis [33]. The detection of EV-B106 from AFP patient having residual weakness at 60 days follow-up is of clinical significance because of its neurotropic behavior and association with poliomyelitis like paralytic disorder. The similar findings by Li et al., (2009), Saskia et al., (2013) and Tan Le et al., (2013) provide further evidence that the viruses other than polio may play an important role in such neurological disorder and need further studies [34-36].

In conclusion, this study underscores the circulation of non-polio AFP enteroviruses with high degree of genetic diversity in FATA. We also suggest investigating the disease burden of non-polio enteroviruses in the country to implement the appropriate public health measures. 
